# LL-VNS attenuates SK2 expression and incidence of arrhythmias following acute myocardial infarction in rats

**DOI:** 10.3389/fcvm.2022.1034888

**Published:** 2023-01-11

**Authors:** Mingxian Chen, Tongjian Zhu, Zhihong Wu, Lin Hu, Zhijian Wu, Qiming Liu, Shenghua Zhou

**Affiliations:** ^1^The Second Xiangya Hospital, Central South University, Changsha, China; ^2^Xiangyang Central Hospital, Xiangyang, Hubei, China

**Keywords:** low-level vagus nerve stimulation, acute myocardial infarction, ventricular arrhythmia, sympathetic nervous activity, SK2

## Abstract

**Objectives:**

Our previous study has demonstrated that low-level vagus nerve stimulation (LL-VNS) protects the heart against ventricular arrhythmias (VAs) induced by acute myocardial infarction (AMI). However, the potential mechanisms by which it influences ventricular electrophysiology remain unknown.

**Materials and methods:**

Forty-five rats were divided into three groups: a Control group (sham AMI followed by sham LL-VNS, *n* = 15), an AMI group (AMI followed by sham LL-VSN for 60 mins, *n* = 15), and an AMI + LL-VNS group (AMI followed by LL-VSN for 60 mins, *n* = 15). Heart rate variability (HRV), ventricular effective refractory period (ERP), ventricular fibrillation threshold (VFT), and left stellate ganglion (LSG) activity were measured at baseline and during AMI. Finally, myocardial tissues were collected for tissue analysis.

**Results:**

AMI directly induced hyperactivity in the LSG and reduced vagal tone as indexed by HRV. AMI also decreased VFT, and shortened ERP but increased ERP dispersion. AMI resulted in an increase in expression of ventricular small-conductance Ca^2+^-activated K^+^ (SK2). However, LL-VNS significantly mitigated or eliminated the effects of AMI.

**Conclusion:**

LL-VNS altered the electrophysiological properties of the ventricles through inhibition of cardiac sympathetic nervous activity and reduction in SK2 expression.

## Graphical Abstract

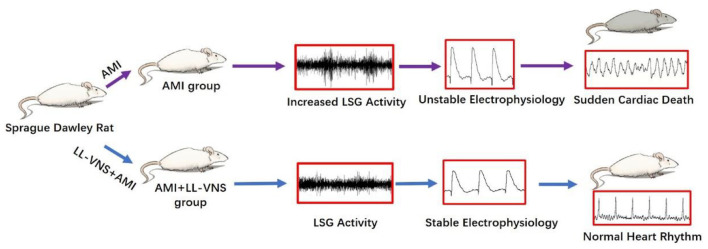

## 1. Introduction

Acute myocardial infarction (AMI) is one of the most severe cardiac diseases, with high morbidity and mortality rates (1). Ventricular arrhythmias (VAs) function as a marker of enhanced risk of sudden cardiac death in AMI. Early rapid reperfusion following coronary artery occlusion remains the most effective strategy to rescue ischemic myocardium and improve long-term clinical outcomes (2). However, reperfusion can also induce sustained ventricular tachycardia, leading to ventricular fibrillation (3). Thus, there is a strong need for a strategy that can effectively decrease the incidence of ventricular arrhythmias caused by ischemia. It is already well-known that autonomic imbalance contributes to the pathogenesis of myocardial ischemia (4). Recently, non-pharmacological neuromodulations have been applied for the prevention of VAs (5). Our previous study has demonstrated that low-level vagus nerve stimulation (LL-VNS) can effectively reduce episodes of ventricular arrhythmia (6). However, the potential mechanisms by which it influences the electrophysiological properties of the ventricles remain unknown.

Ventricular electrophysiological remodeling plays an important role in the genesis of VAs. Recent studies have demonstrated that activation of small-conductance Ca^2+^-activated K^+^ (SK) may be pro-arrhythmic in the heart. Heart failure increases SK2 expression and causes changes in ventricular electrophysiology (7–9), while SK channel blockade prevents arrhythmias. Thus, this has been regarded as a new target of anti-arrhythmic therapy. However, it remains unknown whether LL-VNS exerts anti-arrhythmic effects by regulating SK channel expression. This study explored the underlying mechanisms by which LL-VNS affects VAs. We hypothesized that LL-VNS reduces VAs by modulating expression of the SK channel.

## 2. Materials and methods

### 2.1. Preparation of animals

The protocols and procedures employed in the experiment conducted in this study were approved by the Animal Ethics Committee and the Animal Care and Use Committee of Renmin Hospital of Wuhan University. The standards of the National Institutes of Health (NIH Publication, revised 2011) for the care and use of laboratory animals served as guidelines for all experimental steps. All Sprague Dawley rats (male, weighing 219–299 g) were provided by Hunan SJA Laboratory Animal Co., Ltd. All animals were reared at the Department of Animals for Scientific Research, Renmin Hospital of Wuhan University, in an environment determined to be pathogen-free, with regulated temperature and humidity. During the acclimation period (1 week) before the study started, all animals were housed in groups in a 12:12 h light:dark environment with food and water available.

### 2.2. Experimental protocol and acute myocardial infarction procedure

**FIGURE 1:**
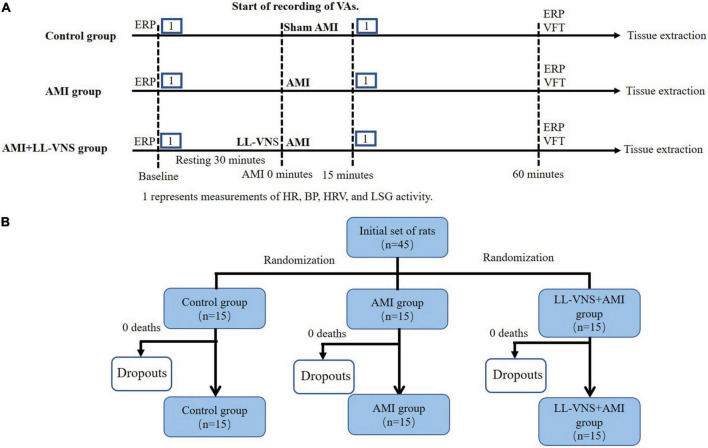
The experimental protocol **(A)** and allocation to groups **(B)**. AMI, acute myocardial infarction; BP, blood pressure; ERP, effective refractory period; HR, heart rate; HRV, heart rate variability; LL-VNS, low-level vagus nerve stimulation; LSG, left stellate ganglion.

SD rats were randomly divided into three groups: a Control group (*n* = 15), an AMI group (*n* = 15), and an LL-VNS + AMI group (*n* = 15) [details in Figure 1A]. All animals were anesthetized *via* intraperitoneal injection of 30 mg/kg 3% sodium pentobarbital. A positive pressure, constant volume rodent ventilator (Anhui Zheng Hua Biological Instrument Co., Ltd., Anhui, China) was used to intubate and ventilate all experimental rats. Throughout the entire experiment, electrocardiogram (ECG) was continuously recorded *via* needle electrodes, which were subcutaneously inserted into the limbs of the rats. The ECG data were collected and analyzed using a Power Lab data acquisition system (AD Instruments, Bella Vista, Australia). In order to measure systemic arterial blood pressure, a catheter was placed into the right carotid artery. Additionally, in order to establish an AMI model, the left anterior descending (LAD) coronary artery was dissected and ligated with a 6-0 prolene suture after thoracotomy at the fourth intercostal space. ST elevation and T-wave changes are hallmarks of a successful AMI model. Rats in the Control group underwent a surgical procedure identical to that administered to the rats in the other groups, however the only difference was that the LAD coronary artery was simply threaded rather than being ligated. In the AMI group, following baseline measurements, the AMI model was established; this was followed by sham LL-VNS for 1 h. The LAD was also ligated in rats in the LL-VNS + AMI group, and LL-VNS was then applied for 1 h. Heart rate and blood pressure were each measured at baseline and 15 min after AMI induction.

### 2.3. Low-level vagus nerve stimulation

The cervical vagal trunk on the left side was exposed and isolated. The cervical vagal trunk was stimulated using a pair of Teflon-coated silver wires (0.1 mm diameter). The left cervical vagal nerve was stimulated with electrical rectangular pulses of 0.1 ms duration at 10 Hz using a stimulator (Grass-S88, Astro-Med, West Warwick, RI, USA). The threshold voltage was defined as the voltage necessary to slow the sinus rate or to reduce atrioventricular conduction. LL-VNS was defined as the application of voltage 80% below the threshold for lowering the heart rate. The actual electrical voltage applied was between 3 and 7 V. LL-VNS began 1 min prior to LAD ligation and continued for 60 min following it (6) (timeline details are provided in Figure 1B).

### 2.4. Measurement of neural activity in the left stellate ganglion

Left stellate ganglion (LSG) activity was recorded for 1 min in each group. The fascia of the LSG was inserted into a tungsten-coated microelectrode, and the chest wall was connected to a group lead. A Power Lab data acquisition system (8/35, AD Instruments, New South Wales, Australia) and an amplifier (DP-304, Warner Instruments, Hamden, CT, USA) were used to record LSG-generated electrical signals. Bandpass filters (from 300 Hz to 1 kHz) and amplification (from 30 to 50 times) were set up prior to each recording. The frequency and amplitude of neural activity, defined as deflections with a signal-to-noise ratio > 3:1, were recorded. LSG activity was measured at baseline and 15 min after AMI induction.

### 2.5. Ventricular effective refractory period measurement

In order to measure ventricular ERP, a specially constructed Ag-AgCl catheter was positioned at three different locations: the ischemic area (under the ligation site, close to the left ventricular apex), the left non-ischemic area, and the right non-ischemic area. Eight successive stimuli S1–S1 were applied with a cycle length of 150 ms; these were followed by pairs of stimuli with a gradually shortened interval preceding an extra S2 until failure of ventricular capture. The time interval between S1 and S2 started at 120 ms and was gradually shortened by 10 ms each time until loss of capture, and then by 1 ms. The longest S1–S2 time interval that failed to lose ventricular capture was taken as a measure of ERP, as described previously (10). ERP was measured at baseline and 60 min after AMI.

### 2.6. Ventricular fibrillation threshold measurement

The right ventricle surface was placed into paired stimulating electrodes to carry out a burst stimulation (4 ms pulse width at 50 Hz). The electrodes were subsequently fixed with a shelf. Each stimulus lasted for 5 s, and a 2-s interval occurred between each consecutive stimulus. The VFT was identified as the lowest voltage that caused sustained ventricular fibrillation (≥ 3 s). The stimulating voltage began at a prespecified level of 4 V and was gradually increased by 1 V, as described previously (11). VFT was measured 60 min after induction of AMI.

### 2.7. Heart rate variability measurement

Chart 8.1 software was used for HRV analysis. The 5-min ECG recording segments taken at baseline and 15 min after AMI were selected for analysis of spectral power, as a measure of HRV, *via* the autoregressive algorithm. Power spectral variables measured were the low-frequency component (LF, between 0.20 and 0.75 Hz), the high-frequency component (HF, between 0.75 and 2.5 Hz) and the ratio of LF to HF (LF/HF). Normalized units are used to express the values of the LF and HF components.

### 2.8. Observation of ventricular arrhythmias

Episodes of VA were recorded during the myocardial ischemic period following AMI in each group. These observations included: number of ventricular premature contractions; number and duration of instances of ventricular tachycardia; and incidence of VF, as previously described in detail (6). Episodes of VA were monitored during the entire AMI period.

### 2.9. Protein preparation and immunoblotting

After measurement of ERP 60 min after induction of AMI, the heart tissues were extracted for tissue analysis. Total SK2 protein expression was detected *via* immunoblot analysis, as described previously (12). After recording of the electrophysiological properties of the ventricles, the ischemic area of the left ventricle was immediately frozen in liquid nitrogen and stored at −80°C. Proteins were separated using SDS-PAGE with 15% polyacrylamide gels. The proteins from the gels were transferred onto PVDF membrane after electrophoresis with SDS-PAGE. The blocking buffer membrane was incubated for 60 min at room temperature and then washed three times for 5 min. After blocking and washing, the blot was incubated overnight at 4°C in a dilute solution (1:1000) of anti-SK2 antibody (Servicebio, Wuhan, China) and mouse monoclonal to β-actin (Abcam, Cambridge, UK). The blots were washed three times following primary antibody incubation, and were subsequently incubated for 60 min with goat anti-rabbit IgG conjugated with horseradish peroxidase. Chemiluminescence was used to measure proteins on Western blots. Video densitometry was used to quantify the signals; the intensities of the bands are expressed relative to β-actin.

### 2.10. RNA-qPCR assay

Real-time PCR assay of SK2 was performed in the same way as in a previously described study (13). For quantification of SK2 mRNA levels, total RNA was extracted *via* homogenization with TriReagent (Ambion, TX, USA). GAPDH was used as a reference gene for semiquantitative measurement. A segment of rat SK2 cDNA sequence was targeted with forward primer 5′-AAC GCA GCC GCC AAT GTA C-3′, reverse primer 5′-CGC TTG GTC ATT CAG TTT CC-3’, while a segment of rat GAPDH sequence was amplified with upstream primer 5′-TGGTATCGTGGAAGGACTCAT-3′ and downstream primer 5′-GTGGGTCGCTGTTGAAGTC-3′. The following conditions were applied for qPCR: 50°C for 2 min, 95°C for 10 min, and 40 cycles of 95°C for 15 s then 60°C for 60 s on an Applied Biosystems 7300HT thermocycler. All samples were run in triplicate, and PCR was repeated twice. The 2^–ΔΔCq^ method, applied using the Relative Expression Software Tool, was used to measure relative expression.

### 2.11. Statistical analysis

Data were analyzed using the SPSS software package, version 22.0 (SPSS, Inc., Chicago, IL, USA). All continuous variables are reported in the form of mean ± SD. Differences between groups were examined using one-way ANOVAs, *t* tests, or two-way repeated-measures ANOVAs. The Kruskal–Wallis test was used to compare the incidence and the duration of VAs among groups; if a significant difference between groups was identified, *post hoc* pairwise comparisons were subsequently carried out using the Dunn multiple comparison test. For count data (i.e., the incidence of VF), Fisher’s exact test was performed to compare the groups. *P* < 0.05 was taken as the threshold for significance.

## 3. Results

There were no significant changes in LL-VNS threshold, indicating that stimulation did not damage the vagal nerve over the course of the experiment.

### 3.1. Effects of LL-VNS on heart rate and arterial blood pressure

As shown in Table 1, there were no significant differences among the three groups in HR at baseline. Although HR after AMI was slightly elevated in the AMI group and the AMI + LL-VNS group, no significant difference was observed between either of these groups and the Control group. In the AMI group, systolic and diastolic blood pressure were significantly lower after AMI compared with the group baseline (*P* < 0.05 in both cases). In the AMI + LL-VNS group, no obvious changes were observed in either systolic or diastolic blood pressure after AMI compared with the group baseline. This indicates that AMI may not induce significant changes in heart rate, but may contribute to a decrease in blood pressure in the rat model of AMI. LL-VNS also did not induce changes in heart rate, but was found to eliminate the effects of AMI on blood pressure.

**TABLE 1:** Effects of low-level vagus nerve stimulation (LL-VNS) on physiological parameters.

	Heart rate (bpm)		Systolic BP (mm Hg)		Diastolic BP (mm Hg)	
	Baseline	After AMI	Baseline	After AMI	Baseline	After AMI
Control group	404 ± 31	409 ± 28	121 ± 14	119 ± 16	93 ± 10	92 ± 9
AMI group	401 ± 34	419 ± 34	123 ± 15	107 ± 14*	93 ± 10	77 ± 8*
AMI + LL-VNS group	402 ± 39	417 ± 40	120 ± 14	112 ± 11	95 ± 12	87 ± 12

Heart rate and blood pressure (BP) were measured at baseline and 15 min after AMI in all three groups. Values are presented in the form of mean ± SD. *n* = 15 per group. *P < 0.05 in a comparison to the group baseline.

### 3.2. Effects of LL-VNS on ventricular ERP, ERP dispersion, and VFT

**FIGURE 2:**
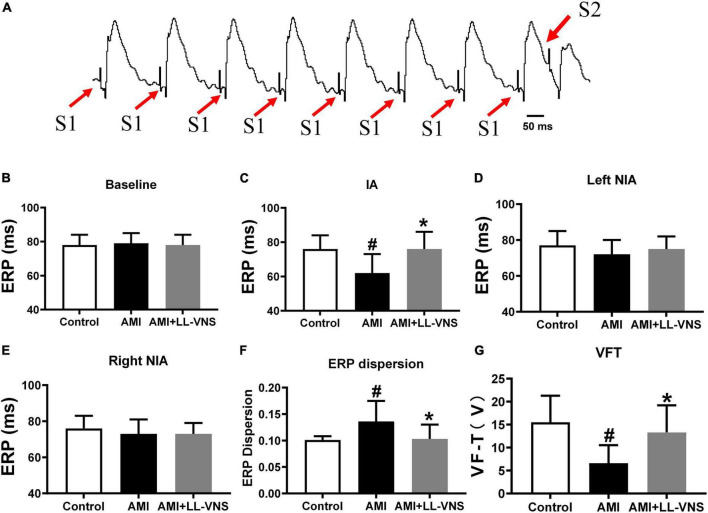
Effects of LL-VNS on the electrophysiological properties of the ventricles. **(A)** Typical example of S1–S2 stimulation scheme in the measurement of ventricular ERP. **(B)** No significant differences among the three groups were observed at baseline. **(C)** LL-VNS prolonged ischemia-induced ERP measured at the ischemic area (IA). **(D,E)** No significant differences were observed among the three groups in ERP measured at the left or right non-ischemic area. **(F)** LL-VNS significantly reduced ischemia-induced ERP dispersion. **(G)** LL-VNS significantly mitigated ischemia-induced reduction in VFT. AMI, acute myocardial infarction; ERP, effective refractory period; IA, ischemic area; LL-VNS, low-level vagus nerve stimulation; NIA, non-ischemic area; VFT, ventricular fibrillation threshold. *n* = 15 per group. #*P* < 0.01 in a comparison with the Control group; *P < 0.01 in a comparison with the AMI group.

Figure 2A shows a schematic diagram of S1-S2 stimuli during ERP measurement. As illustrated in Figure 2B, no significant difference in ERP was found among the three groups at baseline. As shown in Figure 2C, ERP as measured at the ischemic area was significantly reduced in the AMI group compared with the Control group (*P* < 0.05). However, LL-VNS treatment was associated with increased ERP in the ischemic area compared with that of the AMI group (*P* < 0.05). As illustrated in Figures 2D, E, there were no significant differences in ERP as measured at either the right or the left non-ischemic area between the AMI group and the Control group. Meanwhile, there was also no difference between the LL-VNS and AMI groups in ERP measured at the right or left non-ischemic area. As shown in Figures 2F, G, AMI induced an increase in ERP dispersion compared to the Control group, but LL-VNS was associated with reduced ERP dispersion and increased VFT in comparison to the AMI group (*P* < 0.05 in both cases). These results indicate that LL-VNS could stabilize ventricular electrophysiology.

### 3.3. Significant suppression of LSG neural activity in LL-VNS

**FIGURE 3:**
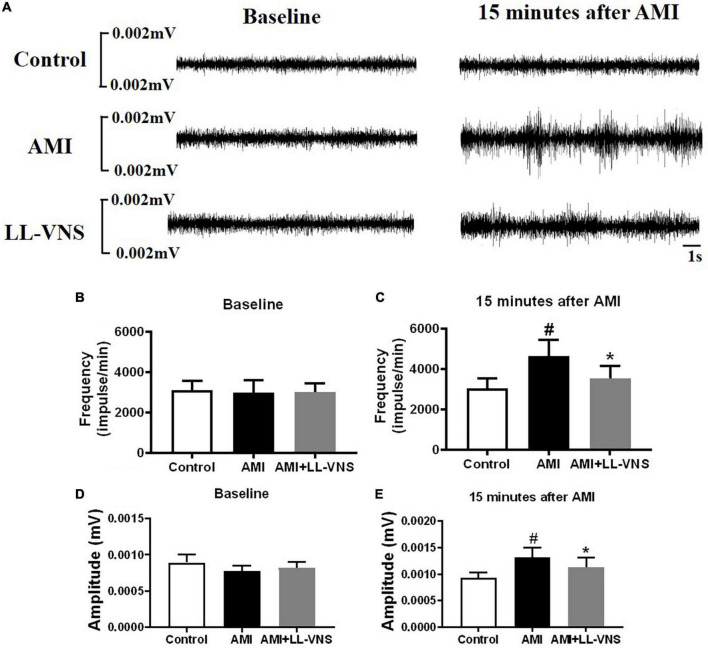
Effects of LL-VNS on neural activity in the left stellate ganglion (LSG). **(A)** Representative examples of LSG neural activity in the Control group, AMI group, and AMI + LL-VNS group. **(B–E)** Quantitative analysis of LSG neural activity in the three groups. LL-VNS significantly mitigated the AMI-induced increases in amplitude and frequency of LSG neural activity. AMI, acute myocardial infarction; LL-VNS, low-level vagus nerve stimulation. *n* = 15 per group. #*P* < 0.05 in a comparison with the Control group. *P < 0.05 in a comparison with the AMI group.

Figure 3A provides representative examples of recordings of LSG neural activity at different points in all three groups. As compared to the Control group, there was a significant increase in both the frequency and the amplitude (Figures 3C, E) of LSG neural activity in the AMI group after AMI. Furthermore, compared to the AMI group, the increase in LSG neural activity induced by AMI was significantly reduced in both frequency and amplitude by LL-VNS. LSG activity directly reflects cardiac sympathetic activity. As illustrated in Figure 3, these results indicate that LL-VNS suppressed cardiac sympathetic activity.

### 3.4. Effects of LL-VNS on HRV

**FIGURE 4:**
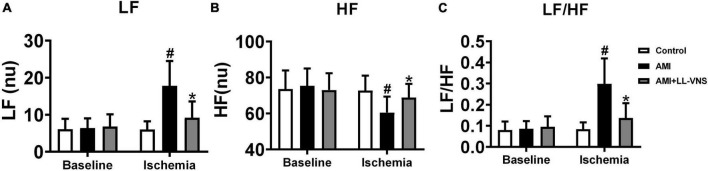
Effects of LL-VNS on cardiac autonomic nervous activity. Panels illustrate changes among the three groups between baseline and measurements taken 30 min after AMI in LF **(A)**, HF **(B)**, and LF/HF **(C)**. AMI induced an increase in LF and LF/HF, but a reduction in HF, as indicated by comparison between the AMI and Control groups. In comparison with the AMI group, LF and LF/HF were significantly reduced in the LL-VNS group, but HF was increased. AMI, acute myocardial infarction; HF, high frequency; LF, low frequency; LF/HF, low frequency to high frequency ratio; LL-VNS, low-level vagus nerve stimulation. *n* = 15 per group. #*P* < 0.05 in a comparison with the Control group. *P < 0.05 in a comparison with the AMI group.

Heart rate variability (HRV) provides indirect insight into the autonomic state of the entire body. The HF component is widely believed to reflect parasympathetic nerve activity, while the LF/HF ratio reflects sympathetic activity. No significant differences among the three groups were observed in LF (Figure 4A), HF (Figure 4B), or LF/HF (Figure 4C) at baseline. Following AMI, LF and LF/HF were significantly elevated and HF was markedly decreased in the AMI group (*P* < 0.05). LL-VNS led to a significant decrease in LF and LF/HF (*P* < 0.05), but no significant increase in HF. Based on this result, AMI induced an increase in sympathetic activity and a decrease in parasympathetic activity. Therefore, these findings indicate that AMI causes autonomic imbalance, but LL-VNS could maintain autonomic balance.

### 3.5. Effects of LL-VNS on the occurrence of VAs

**FIGURE 5:**
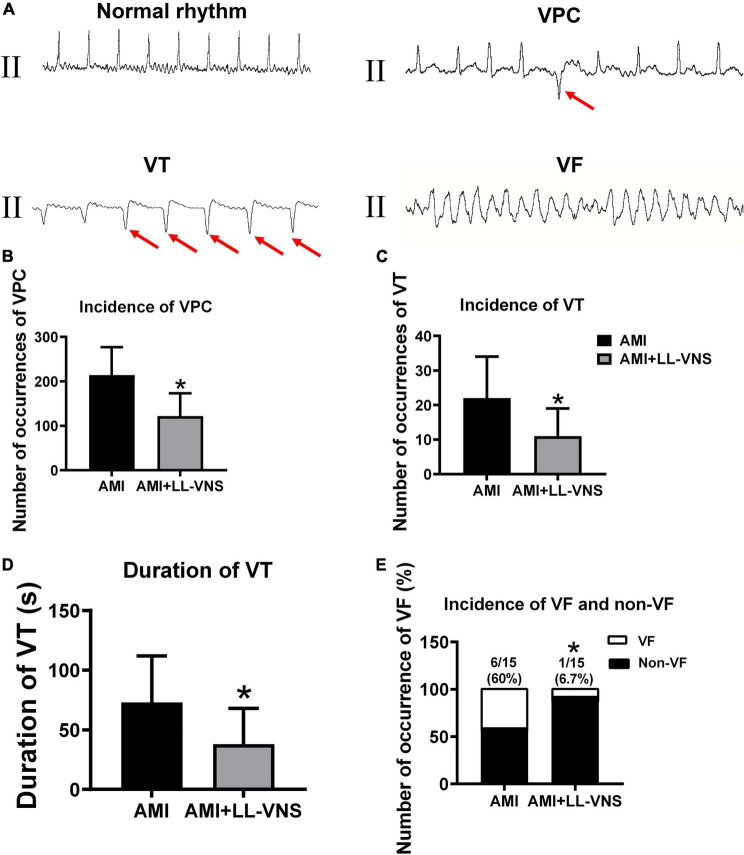
The occurrence of ventricular arrhythmias after complete occlusion of the left anterior descending branch. **(A)** ECG traces representing typical examples of VAs, including VPC, VT, and VF. **(B)** Comparison of the incidence of VPC throughout the entire 60 min between the AMI group and the AMI + LL-VNS group. **(C)** LL-VNS decreased the incidence of VT within 60 min after AMI. **(D)** LL-VNS shortened the duration of episodes of VT occurring within 60 min after AMI. **(E)** LL-VNS reduced the incidence of spontaneous VF. AMI, acute myocardial infarction; ECG, electrocardiogram; LL-VNS, low-level vagus nerve stimulation; VF, ventricular fibrillation; VPC, ventricular premature contraction. *n* = 15 per group. *P < 0.05 in a comparison with the AMI group.

The ECG was continuously monitored for 1 h to detect the occurrence of VAs. Representative examples are provided in Figure 5A. No ventricular arrhythmic episodes occurred in the Control group, whereas VPC or VT occurred in all experimental animals in the AMI group. However, LL-VNS treatment reduced the occurrence of VPC and VT. As illustrated in Figure 5B, LL-VNS significantly reduced the incidence of VPC compared with the AMI group (*P* < 0.05). As illustrated in Figures 5C, D, LL-VNS also significantly reduced the incidence of VT and produced a clear reduction in the duration of VT in comparison with the AMI group (both *P* < 0.05). Additionally, as shown in Figure 5E LL-VNS significantly reduced the incidence of spontaneous VF in comparison with the AMI group (*P* < 0.05). Spontaneous VF occurred in six rats in the AMI group, but in only one rat in the AMI + LL-VNS group. After the appearance of VF in any of the rats, heart compression was administered; all of the rats were successfully rescued.

### 3.6. Effects of LL-VNS on SK2 expression

**FIGURE 6:**
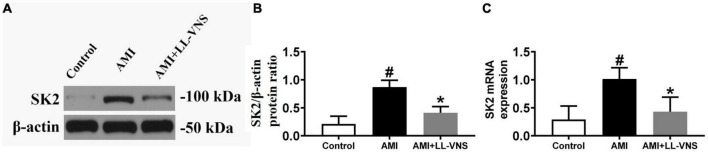
Effects of low-level vagus nerve stimulation (LL-VNS) on SK expression induced by acute myocardial infarction (AMI). **(A)** Representative immunoblots of ventricular SK2. **(B)** Immunoblot analysis of ventricular SK2 protein expression. AMI contributed to a significant increase in ventricular SK2 protein expression; however, LL-VNS clearly reduced ventricular SK2 protein expression by comparison. **(C)** qRT-PCR analysis of ventricular SK2 mRNA expression. AMI induced an increase in SK2 mRNA expression; this was reduced in the LL-VNS group. *n* = 15 per group. #*P* < 0.05 in a comparison with the Control group. *P < 0.05 in a comparison with the AMI group.

Figure 6A shows a representative immunoblot prepared with anti-SK2 antibody. As illustrated in Figure 6B, AMI significantly increased SK2 protein expression in comparison with the Control group. LL-VNS significantly reduced the expression of ventricular SK2 in the ischemic area in comparison with the AMI group (*P* < 0.05). Figure 6C shows the effects of LL-VNS on ventricular SK2 mRNA expression. AMI increased SK2 expression in comparison with the Control group, while LL-VNS mitigated the AMI-induced increase in ventricular SK2 mRNA expression in the ischemic area in comparison with the AMI group.

## 4. Discussion

### 4.1. Major findings

The aim of the present study was to evaluate the potential mechanisms underlying the alteration of ventricular electrophysiology induced by LL-VNS in rat models. The significant findings were as follows: (1) LL-VNS may prolong ventricular ERP, maintain ERP dispersion, and increase VFT; (2) LL-VNS was found to increase parasympathetic tone, but was associated with reduced sympathetic activity; and (3) LL-VNS altered ventricular electrophysiology *via* reduction of ventricular SK2 expression.

### 4.2. Cardiac autonomic nervous system dysfunction and ventricular electrophysiology

Ventricular arrhythmias (Vas) have been proposed as a marker for increased risk of sudden death (14). Ventricular tachycardias commonly occur early in ischemia, and raise the rate of complications from 10 to 40% (15). Cardiac autonomic nervous dysfunction associated with sympathetic hyperactivity and vagal withdrawal has been recognized as a critical trigger in the generation of ventricular arrhythmias in the acute phase of myocardial infarction (16). Afferent-mediated activation caused by acute myocardial infarction increases sympathetic drive and reduces vagal tone (17). The overactivation of sympathetic tone leads to a suitable substrate for maintenance of the propagation of VAs. This substrate can take the form of structural and/or electrophysiological remodeling. Activation of the sympathetic nervous system increases the release of catecholamine. In turn, catecholamine combines with β-adrenergic receptors and subsequently results in afterdepolarization and abnormal automaticity. Stimulation of the β-adrenergic receptor increases release of Ca^2+^ from the sarcoplasmic reticulum, producing Ca2 + overload, and ultimately generates an afterdepolarization. Remodeling of ion channels induced by sympathetic nervous system activity in the acute phase of myocardial infarction can influence action potential duration, inducing ERP changes and predisposal to an arrhythmic trigger (18) independently of heart rate. In the present study, we found that AMI induced a reduced ERP and this was followed by overactivation of sympathetic tone. However, LL-VNS treatment resulted in increased ERP and suppressed sympathetic activity in comparison with the AMI group. These findings indicate that LL-VNS could balance cardiac autonomic dysfunction and stabilize the electrophysiological properties of the ventricles. Therefore, LL-VNS in the absence of any change in heart rate could be targeted as an alternative therapeutic approach to reduction of the risk of VAs.

### 4.3. LL-VNS maintains cardiac autonomic nervous function *via* afferent vagal nerve activity

Previous studies have reported that vagus nerve stimulation (VNS) could protect the heart against VAs following heart rate reduction (19, 20). VNS has been found to regulate autonomic dysfunction *via* efferent terminals. However, in the present study, LL-VNS was found to regulate cardiac autonomic nervous activity in the absence of any heart rate change. This finding indicates that LL-VNS modulates autonomic function *via* afferent activity. The stimulation electrode is affiliated in the left-side vagal nerve and delivers a biphasic current that continuously cycles between on and off periods. Vagal afferent activation, generated by the VNS, projects to the medulla located in the brainstem. The medulla contains cell bodies of the sympathetic and parasympathetic nervous system. The nucleus tractus solitarius (NTS) of the medulla receives vagal afferent input and integrates the information. Neural connections from the NTS activate the sympathetic neurons located in the rostral ventrolateral medulla (RVM), and inhibit the parasympathetic neurons located in the dorsal vagal nucleus (DVN) and nucleus ambiguous (NA). Not only does VNS appear to increase vagal tone in the heart, it may also decrease sympathetic activity to some extent.

### 4.4. LL-VNS modulates ventricular Electrophysiology, potentially *via* SK2 expression

Several studies have demonstrated that an increase in ventricular SK2 occurs in pathophysiological conditions, including heart failure and chronic myocardial infarction, resulting in a reduction in ERP (21, 22). SK channels are expressed in the ventricles; it is possible that AMI may open SK channels and thereby induce ventricular repolarization. Recent studies have reported observing pro-arrhythmic properties of the SK channel in rats undergoing induced AMI. Inhibition of SK channels could reduce the VT, as well as completely eliminating VF. Previous work has indicated that SK channel blockade during AMI protects against AMI-induced arrhythmia and VF (23). In our study, we found that AMI induced overexpression of SK2. However, following LL-VNS treatment, the increase in SK2 expression induced by AMI was eliminated. This indicates that LL-VNS potentially suppresses SK2 overexpression. In summary, the results of this study suggest that LL-VNS inhibits ventricular arrhythmias induced by AMI, and that it potentially does so by modulating the expression of SK2 protein. However, the detailed mechanisms underlying the effect of LL-VNS on SK2 expression require further exploration.

### 4.5. Clinical implications

The occurrence of VAs increases the risk of mortality among patients with AMI. How to effectively prevent and suppress VAs following AMI is therefore among the most pressing issues in this domain. In the present study, we have provided experimental evidence that LL-VNS is an effective non-pharmaceutical strategy for the prevention of VAs during the acute phase of myocardial infarction. According to the results of this study, the possible mechanisms underlying this effect may be associated with the reduction in cardiac sympathetic activity and increase in cardiac vagal activity which follow a reduction in SK2 expression. In clinical settings, vagus nerve stimulation has been applied in treatment of heart failure, depression, and Parkinson’s syndrome. Therefore, the present study further indicates that LL-VNS may serve as a promising potential option in the prevention or reduction of VAs during AMI.

### 4.6. Study limitations

There are several limitations to the present study. First, the incidence of spontaneous VF induced by AMI was too low during the acute phase; however, we stimulated the ventricular surface and measured VF threshold instead. Second, we did not measure vagal activity directly because of the small size of the GP; however, we measured HRV instead, as a reflection of vagal activity. Third, we did not further explore the detailed mechanisms underlying the effect of LL-VNS on SK2 expression. Fourth, the use of anesthesia could potentially have masked the influence of VNS on heart rate and blood pressure. However, all rats were anesthetized *via* the same procedure. Further investigation is required to explore the potential effects of VNS on the heart in isolation. Finally, only the short-term effects of LL-VSN on AMI-induced VAs were investigated in the present study, while the long-term benefits remain unknown.

## 5. Conclusion

Low-level vagus nerve stimulation (LL-VNS) could prevent the occurrence of VAs during the acute phase of myocardial infarction. The potential mechanisms might be associated with its balancing effect on cardiac autonomic dysfunction, *via* inhibition of sympathetic nervous activity and activation of vagal tone, as a result of the underlying reduction in ventricular SK2 overexpression.

## Data availability statement

The original contributions presented in this study are included in the article/supplementary material, further inquiries can be directed to the corresponding author.

## Ethics statement

The animal study was reviewed and approved by Wuhan Renmin Hospital.

## Author contributions

MC, TZ, and SZ participated in the study design and drafted the manuscript. ZHW and ZJW contributed to data collection. MC and LH were responsible for writing the manuscript. SZ and QL contributed to the manuscript revision. All authors contributed to the article and approved the submitted version.
